# GOOD NUTRITION PRACTICE

**Published:** 2014

Having the slogan “Good Nutrition Practice”, the 15th Anniversary Edition of the National Symposium of Clinical Nutrition with international participation of the Romanian Society of Enteral and Parenteral Nutrition (ROSEPN), took place in the middle of November 2014, in Poiana Brasov, a blessed Romanian region, characterized by peace and relaxation.

**Fig. 1:**
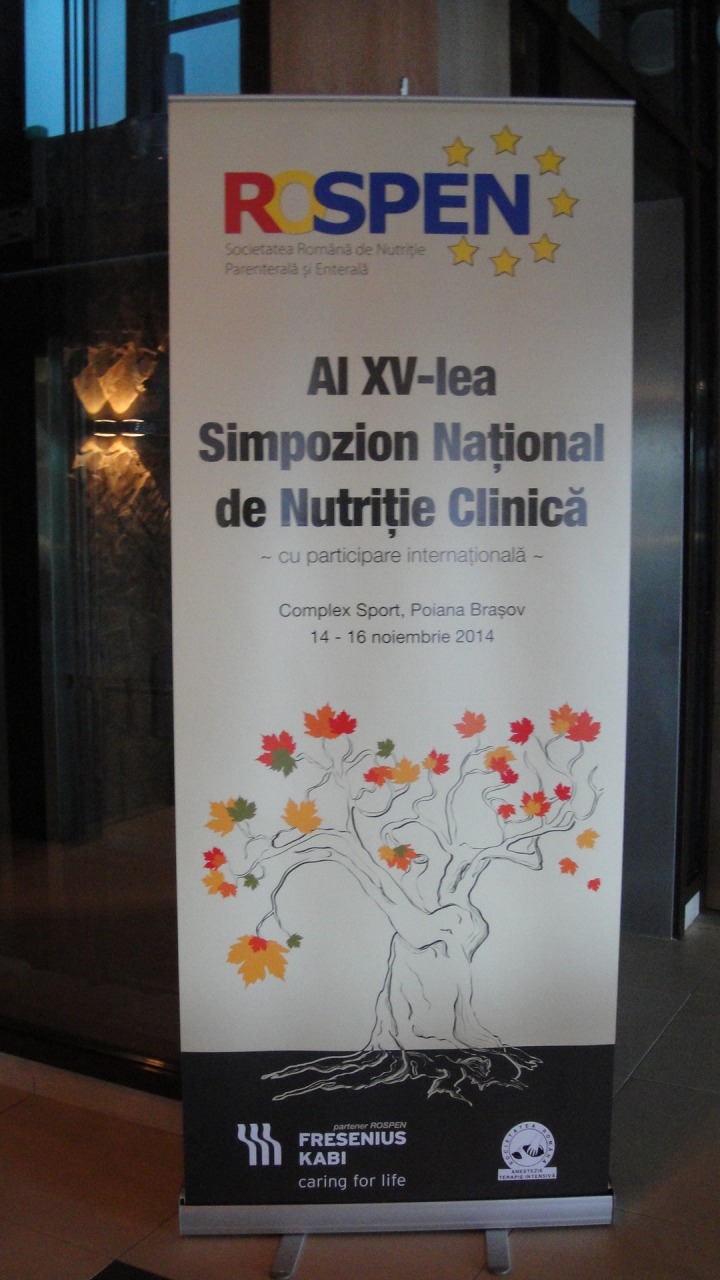
The 15th National Symposium of Clinical Nutrition - poster

Just like Professor Ioana Grintescu, President of ROSEPN affirmed in the opening of the event, the purpose of this edition was to represent the memorable step in presenting some recommendations in the practical realization of some standards in the clinical nutrition so that, by the analysis of each stage and of the actual practices, to identify the progresses made and to propose future development directions, full of dynamics and efficiency. 

**Fig. 2:**
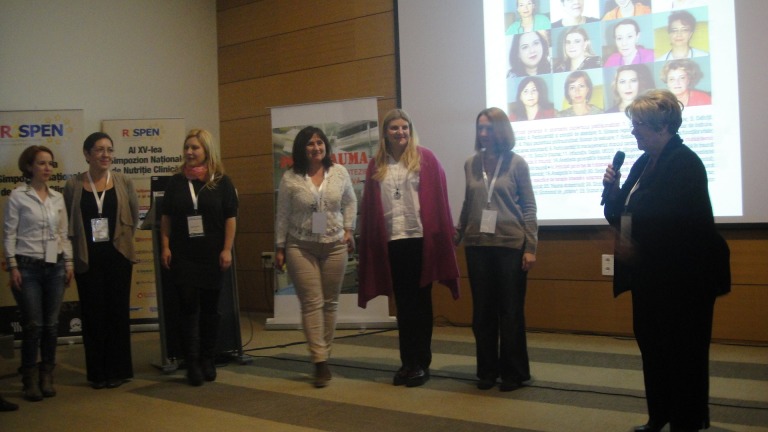
Professor Ioana Grintescu, MD, President of ROSEPN – book presentation

The presentations were followed with great interest by an impressive number of professionals who came to find the novelties in the field, to learn from the other colleagues’ experience and to generously offer bits of their daily experience. 

In addition, presentations such as: “Role of Omega-3-Fatty Acids in the Critically Ill: is there anything new?” by Prof. Reto Stocker, MD; “What is the efficacy of colloids in surgical patients?” by Prof. Dan Longrois, MD; “Energy and protein requirement in the ICU” by Prof. Remy Meier, MD; “Nutrition after total gastrectomy” by Prof. Mircea Beuran, MD; “The value of pletysmography in perioperative fluid therapy” by Prof. Şerban Bubenek, MD, were received with great receptivity by the public.

**Fig. 3:**
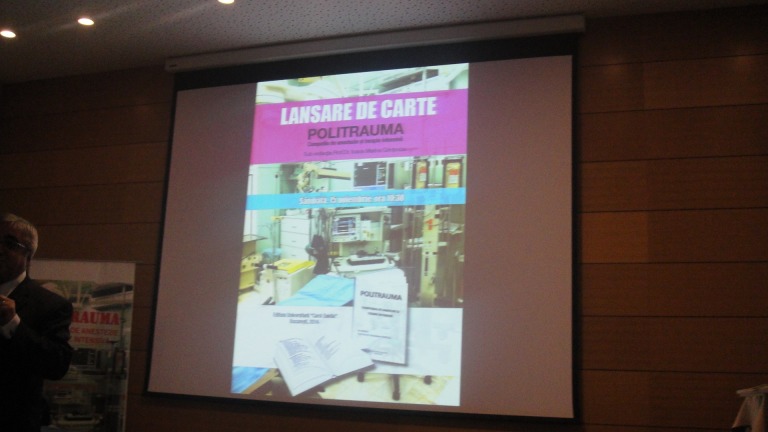
“Politrauma – Compendium of Anesthesia and Intensive Therapy” book launch

**Fig. 4:**
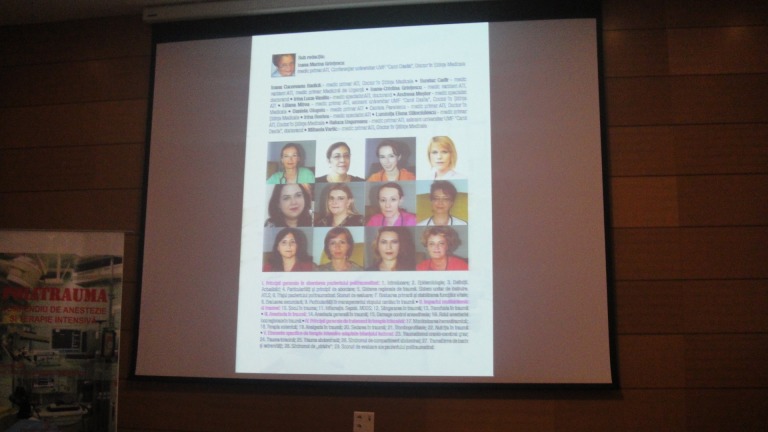
Presentation of the 13 authors who contributed to the realization of the book

There were two events which marked the presentations in the symposium, due to their novelty and originality: the “Microbiotic” Conference, moderated by Prof. Remy Meier, MD, and the book launch “Politrauma – Compendium of Anesthesia and Intensive Therapy”, edited by the prestigious “Carol Davila” University Press and coordinated by Prof. Ioana Maria Grintescu, MD, representing the result of the extraordinary team of the greatest and most solicited emergency hospital in Romania, “Floreasca” Emergency Hospital, a symbol, school and model for the emergency cases in our country. The 13 exceptional and professional authors have found the moment, in their little free time, to write bits of their daily fight for the benefit of life.

**Fig. 5:**
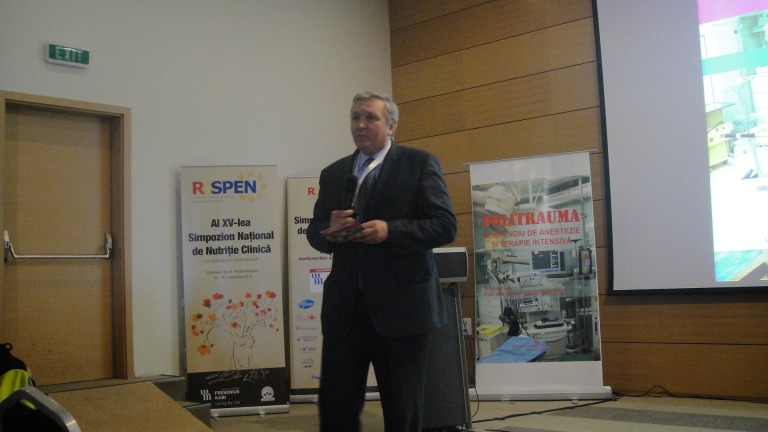
The intervention of Prof. Mircea Beuran, MD, regarding the book launched at the event

 And, maybe, the amount of thoughts expressed on the occasion of this book launching was the best represented by the authorized, professional and spiritual intervention of our excellent surgeon Prof. Mircea Beuran, MD, Head of the Surgery Department in “Floreasca” Emergency Hospital and Director of the Surgery Department in “Carol Davila” University of Medicine and Pharmacy, Bucharest.

**Fig. 6:**
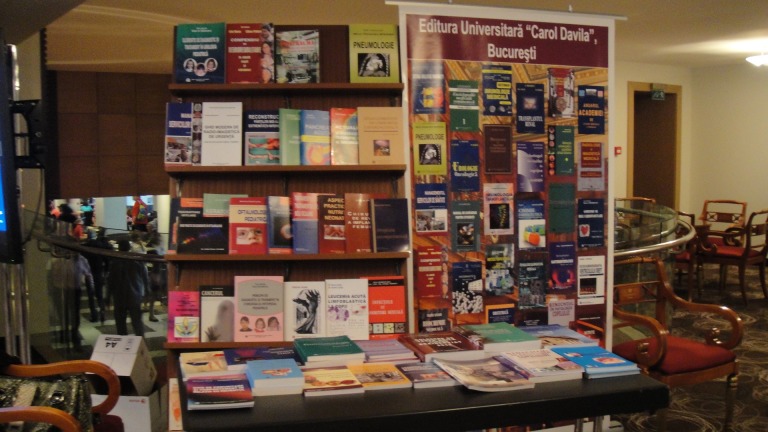
“Carol Davila” University Press books exhibition

During the entire period of the papers presentations, the scientific quality of the presentations, the practical value of the demonstrations and the professional appearance of the medical devices and medical books exhibition have harmoniously linked to the variety and diversity of the program and the wonderful sight of the “Carpathians Pearl”, thanks to the organizers’ efforts. 

**Assoc. Prof. Dr. Eng. VL Purcarea**

